# Personalized peripheral vascular interventional embolization for tumor: tailoring treatment to improve outcomes

**DOI:** 10.3389/fmed.2025.1621148

**Published:** 2025-09-26

**Authors:** Xinyue Qi, Jintai Liu, Tianlong Liu, Huaxin Hao

**Affiliations:** Department of Interventional Medicine, The Third Affiliated Hospital of Gansu University of Chinese Medicine, Baiyin, China

**Keywords:** personalized medicine, tumor embolization, interventional oncology, peripheral vascular disease, drug-eluting beads, tumor biology, therapeutic outcomes, precision treatment

## Abstract

Malignant tumors pose a significant global health burden, necessitating innovative treatment approaches. Personalized peripheral vascular interventional embolization emerges as a promising strategy to enhance outcomes in tumor therapy. This review consolidates evidence on the principles, influencing factors, implementation processes, and clinical applications of this approach. By analyzing tumor vascular anatomy and biological behavior, along with patient-specific factors, clinicians can tailor embolization techniques and materials to maximize efficacy and minimize complications. In practical clinical settings, personalized embolization has shown remarkable potential to enhance patient outcomes. For instance, in patients with hepatocellular carcinoma, personalized chemoembolization not only significantly improved survival rates but also reduced post-procedure complications, thereby improving quality of life. Similarly, in colorectal cancer liver metastases, the combination of embolization with anti-angiogenic agents has proven effective in controlling disease progression, offering a new therapeutic option where conventional treatments fall short. Despite challenges such as technical complexity and treatment costs, advancements in imaging technology, genomics, and novel embolic agent development offer substantial opportunities to refine and expand the application of personalized interventional embolization, potentially transforming the landscape of cancer treatment.

## 1 Introduction

Malignant tumors exact a heavy toll on global health, with cancer being the second leading cause of death worldwide, claiming nearly 10 million lives in 2020 (1). In the United States, approximately 1.9 million new cancer cases were diagnosed in 2023. In China, cancer incidence and mortality rates have been rising over the years, resulting in about 4.06 million new cases and 2.41 million deaths in 2022 (2). Despite advancements in radiation therapy, chemotherapy, targeted therapy, and immunotherapy, these treatments often fall short for advanced malignant solid tumors (3). Patients frequently suffer severe side effects, and tumor heterogeneity makes them respond variably to therapies (4). Consequently, there is an urgent imperative to develop new technologies for personalized treatment (5).

Interventional embolization therapy has emerged as a promising approach in cancer treatment (6, 7). However, most existing reviews have broadly summarized embolization techniques without critically addressing how personalized strategies—based on tumor biology, vascular architecture, and patient-specific factors—can significantly alter clinical outcomes. For example, transarterial chemoembolization (TACE) utilizing drug-eluting beads (DEBs) has demonstrated favorable clinical outcomes (8). DEBs microspheres can be loaded with doxorubicin and have shown clinical efficacy in treating colorectal cancer metastases to the liver (6). HepaSphere microspheres, approved by Conformité Européenne Mark for TACE of hepatocellular carcinoma (HCC) in combination with doxorubicin, can swell to occlude vessels and deliver drugs (9). Additionally, Ding et al. developed alginate-chitosan microspheres loaded with Norcantharidin, which showed significant tumor growth suppression *in vitro* (10). Choi et al. (11) fabricated poly lactic acid-polyglycolic acid (PLGA) microspheres loaded with sorafenib for HCC embolization. In a Renca tumor mouse model, combined cisplatin and sorafenib-loaded microspheres achieved tumor shrinkage, underscoring the potential of interventional embolization therapy.

Emerging evidence suggests that programmed cell death pathways, including PANoptosis—a novel inflammatory form of regulated cell death—may play a pivotal role in modulating tumor immune microenvironment and therapeutic response in HCC. Xiang et al. (12) recently proposed that targeting PANoptosis-related molecular signatures could serve as a promising diagnostic and therapeutic strategy, potentially enhancing the efficacy of interventional embolization by sensitizing tumor cells to ischemia-induced death and modulating immune surveillance. However, due to the heterogeneity of tumors across individuals, a one-size-fits-all approach to treatment is suboptimal. Tumors vary in vascular anatomy, biological behavior, and genetic background, necessitating personalized treatment (13). Research found that patients with HCC receiving personalized chemoembolization exhibited significantly improved survival rates and quality of life compared to conventional treatment (14, 15). Similarly, Vogl et al. (16) revealed that personalized embolization for colorectal cancer liver metastases effectively controlled disease progression and prolonged patient survival.

While existing studies have explored the use of embolization in tumor treatment, our review uniquely consolidates evidence on the principles, influencing factors, implementation processes, and clinical applications of personalized peripheral vascular interventional embolization. By integrating tumor biology, vascular anatomy, and patient-specific factors, this approach offers a more precise and effective treatment strategy compared to conventional methods. This review uniquely synthesizes the latest advancements in personalized embolization, highlighting its potential to overcome the limitations of traditional therapies through tailored treatment strategies. Compared to prior reviews, our work provides a comprehensive, evidence-based framework that links tumor biology, vascular mapping, and patient-specific factors to clinical decision-making in interventional oncology.

## 2 Principles of personalized peripheral vascular interventional embolization for tumors

To achieve such personalized outcomes, a deep understanding of the principles underlying peripheral vascular interventional embolization is essential. Precision in treatment is paramount for effective tumor management, and personalized peripheral vascular interventional embolization exemplifies this approach (17). The principles guiding this method have evolved considerably, driven by a deeper understanding of tumor biology and vascular anatomy (18). Initially, interventional embolization was employed as a palliative measure to alleviate symptoms and control bleeding (19). However, as experience grew and technology advanced, its potential as a curative option became evident. Research demonstrated that the efficacy of chemoembolization could be enhanced by optimizing the size and composition of embolic agents to match tumor vascular characteristics (20, 21). Similarly, Choi et al. (22) highlighted the importance of considering tumor blood flow dynamics and vascular permeability when selecting embolic materials and determining drug doses. These findings highlight the necessity of a personalized approach, wherein treatment strategies are specifically tailored to the unique vascular and biological characteristics of each tumor. The development of these principles marked a shift from a one-size-fits-all model to one that recognizes the unique requirements of different tumors, ultimately aiming to maximize therapeutic outcomes while minimizing complications.

The vascular anatomy of tumors is not only crucial for shaping interventional embolization strategies but also determines the choice of embolic agents and techniques (23). For instance, HCC typically has a hypervascular nature, with abundant arterial blood supply. This characteristic makes HCC amenable to TACE, where embolic agents can be delivered through the hepatic artery to induce tumor ischemia and necrosis (24). A study by Long et al. showed that using smaller embolic particles could more effectively occlude the tumor’s feeding arteries, achieving better tumor shrinkage and improving patient survival rates (18). Conversely, renal cell carcinoma (RCC) often has a more complex vascular network with variability in the number and size of feeding arteries. In such cases, a more precise approach is required (25). Calvo et al. (26) indicated that using a combination of embolic agents with different particle sizes could achieve more complete vascular occlusion, thereby enhancing treatment efficacy. For colorectal cancer liver metastases, the vascular anatomy differs from primary liver tumors, with a more homogeneous distribution of blood flow (22). Here, Choi et al. (22) found that using DEBs loaded with chemotherapy drugs could achieve sustained drug release and more uniform drug distribution within the tumor. This approach not only improved treatment efficacy but also reduced systemic drug toxicity. These examples illustrate how understanding the vascular anatomy of different tumors allows for the selection of appropriate embolic agents and techniques to maximize treatment outcomes.

The biological behavior of tumors also significantly influences interventional embolization strategies (27). Tumors vary in growth rate, invasive capacity, and angiogenesis characteristics, all of which must be considered when planning treatment. Tumors with rapid growth rates, such as pancreatic neuroendocrine tumors (NETs), often have high metabolic demands and are more reliant on neoangiogenesis (27). Research has shown that combining anti-angiogenic drugs with embolization therapy can effectively inhibit tumor growth and improve patient survival (28). For example, Liu et al. (29) demonstrated that using embolic agents loaded with anti-angiogenic drugs could not only block the tumor’s blood supply but also inhibit the formation of new vessels, thereby suppressing tumor growth. Tumors with strong invasive capabilities, such as hepatocellular carcinoma, are more likely to invade surrounding tissues and blood vessels (30). In such cases, the embolization treatment must not only target the tumor itself but also consider the potential impact on surrounding tissues. Nam et al. (31) indicated that using embolic agents with a more controlled release profile could reduce the risk of drug leakage into surrounding tissues, thereby minimizing complications. Tumors with high angiogenesis activity, such as RCC, rely heavily on neoangiogenesis for growth and metastasis (32). Research has shown that combining embolization therapy with anti-angiogenic drugs can effectively inhibit tumor angiogenesis and improve treatment outcomes (33). For instance, a study demonstrated that using embolic agents loaded with anti-angiogenic drugs could not only block the tumor’s blood supply but also inhibit the formation of new vessels, leading to significant tumor shrinkage and improved patient survival (34).

## 3 Factors influencing personalized treatment decisions

**Figure 1:**
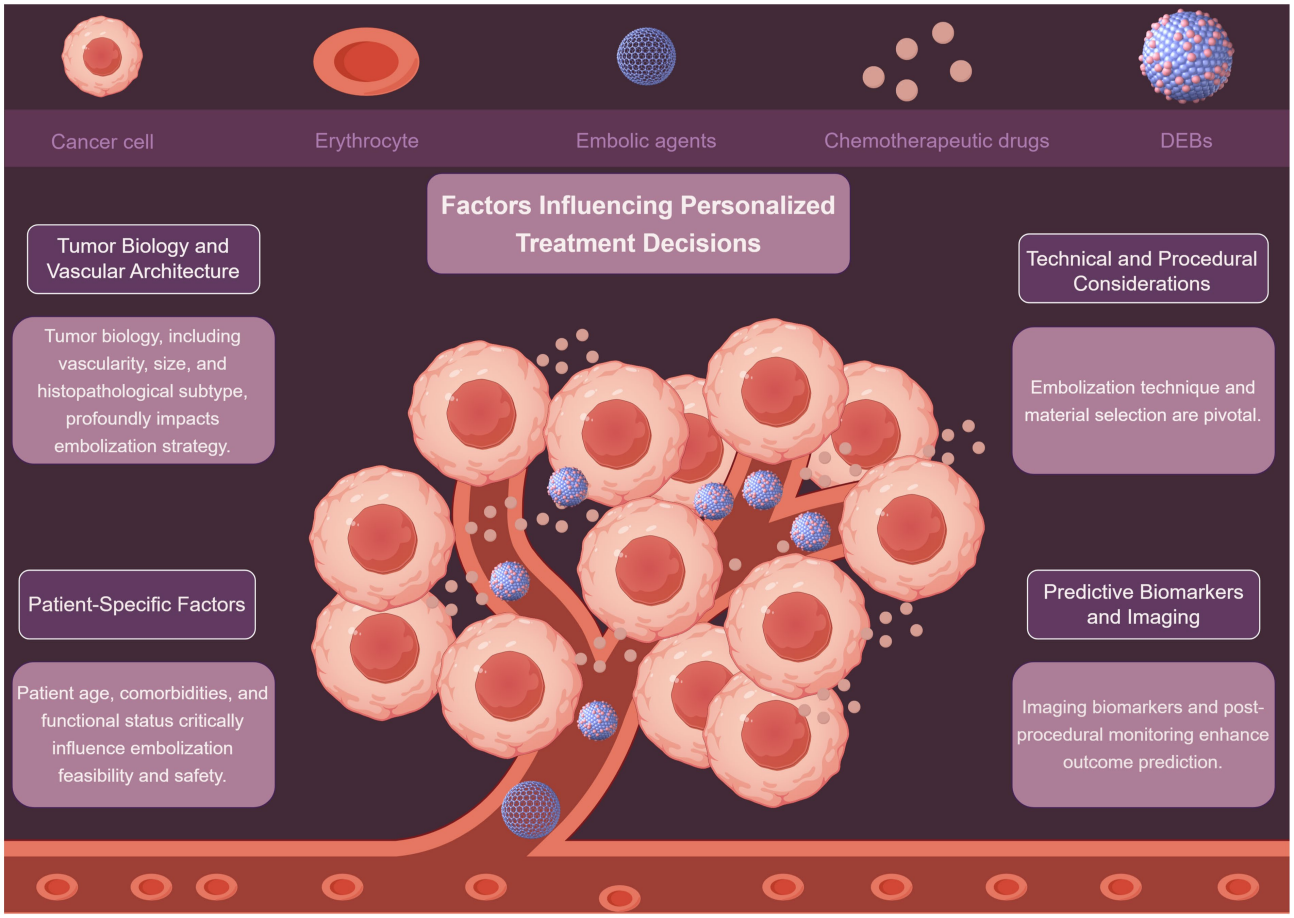
Displaying the factors influencing personalized treatment decisions by Figdraw.

Personalized peripheral vascular interventional embolization for tumors requires a comprehensive decision-making process that integrates multiple factors (Figure 1). These factors include tumor biology, patient-specific characteristics, and technical considerations, among others. A detailed discussion of these factors is presented below.

### 3.1 Tumor biology and vascular architecture

Tumor biology plays a vital role in shaping interventional embolization strategies. Tumors exhibit diverse vascular patterns, and understanding these differences is crucial for effective treatment. Hypervascular tumors, such as renal angiomyolipoma (AML), respond well to embolization, with tumor shrinkage rates correlating with baseline size and vascular density (35, 36). Mechanistically, high vascular density increases the delivery of embolic agents to the tumor site, enhancing ischemia-induced necrosis. Conversely, hypovascular tumors may require adjunctive strategies such as radioembolization or systemic therapy to overcome limited perfusion-mediated drug delivery. Lin et al. (35) demonstrated that AMLs diameter >4 cm and high intratumoral vascularity predicted significant post-embolization volume reduction (OR: 3.2, *p* < 0.01). Conversely, hypovascular metastases (e.g., colorectal liver metastases) may require combination therapies like radioembolization with Yttrium-90 to enhance efficacy (37). Notably, NETs exhibit heterogeneous vascular patterns; Bai et al. (38) found that embolization outcomes varied significantly between NETs subtypes, with pancreatic NETs showing better response than gastrointestinal counterparts (HR: 1.8, *p* = 0.03). This biological heterogeneity underscores the need for pre-procedural imaging biomarkers (e.g., perfusion CT, DCE-MRI) to quantify vascularity and predict embolic agent distribution, enabling a mechanistically informed selection of embolization strategy. While tumor size and vascularity are consistently identified as predictors (35, 36), discrepancies exist in thresholds for “high-risk” tumor size (e.g., 4 cm vs. 5 cm), likely due to differences in embolic agents and follow-up durations. Future studies should standardize vascularity assessment using quantitative imaging biomarkers.

### 3.2 Patient-specific factors

Patient-specific factors also significantly impact the feasibility and safety of embolization. Patient age, comorbidities, and functional status must be carefully evaluated. Arslan and Degirmencioglu (39) identified age >65 years, Child-Pugh B/C cirrhosis, and hypoalbuminemia as independent risk factors for post-embolization syndrome after TACE (AUC: 0.76). From a mechanistic perspective, hypoalbuminemia reflects reduced hepatic synthetic function and increased systemic inflammation, which may impair post-embolization recovery and increase susceptibility to infection or hepatic decompensation. Similarly, patients with ECOG ≥2 often have reduced systemic reserve, limiting their ability to tolerate procedural stress or post-embolization inflammation. Similarly, Kim et al. (40) reported higher complication rates in bladder cancer patients with ECOG ≥2 undergoing superselective vesical artery embolization (OR: 4.1, *p* < 0.001). Conversely, Hongyo et al. (36) emphasized that renal function preservation in AML patients favored selective embolization over surgery, particularly for those with chronic kidney disease (eGFR <60 mL/min/1.73 m^2^). The heterogeneity in comorbidity assessment complicates cross-study comparisons. Notably, while albumin-bilirubin (ALBI) grade predicts Yttrium-90 radioembolization survival in metastatic liver tumors, its utility in non-hepatocellular malignancies remains underexplored.

### 3.3 Technical and procedural considerations

Embolization technique and material selection are pivotal. Liu et al. (41) showed that superselective embolization minimizes non-target ischemia. They combined radiofrequency ablation with embolization for renal AMLs and achieved 92% tumor control with preserved renal function. Mechanistically, superselective catheterization enables targeted delivery of embolic agents to the tumor-feeding vessels while preserving adjacent normal tissue perfusion. This reduces the risk of post-embolization syndrome and organ dysfunction, particularly in patients with limited functional reserve. For gastrointestinal hemorrhage, Xu et al. (42) demonstrated superior hemostasis with Fuaile adhesive compared to gelatin sponge (success rate: 94% vs. 78%, *p* = 0.02). Emerging approaches, like sequential embolization-cryoablation-osteoplasty for osseous tumors, highlight the role of multimodal strategies in complex cases (43).

Despite advances, embolic agent selection remains contentious. Crawford et al. (44) noted no significant difference in benign liver tumor outcomes between particles and lipiodol-based regimens, suggesting agent choice should align with operator expertise. However, particle size impacts distal penetration; smaller particles (<100 μm) may increase PES risk4, necessitating personalized sizing. Importantly, the interaction between particle size and vascular resistance is a key determinant of embolic distribution. Smaller particles penetrate deeper into the tumor microvasculature but may also pass into collateral or shunt vessels, increasing the risk of non-target embolization. Thus, a mechanistic understanding of tumor hemodynamics (e.g., arteriovenous shunting, pressure gradients) is essential for safe and effective agent selection.

### 3.4 Predictive biomarkers and imaging

Imaging biomarkers and post-procedural monitoring enhance outcome prediction. Tsai et al. (45) developed a cone-beam CT-based lipiodol deposition scoring system to predict 1-year HCC response post-TACE (AUC: 0.88). Similarly, Azar et al. (37) validated ALBI grade as a prognostic marker for Yttrium-90 radioembolization in non-HCC liver malignancies (median OS: 15.2 vs. 8.1 months for ALBI grade 1 vs. 2, *p* = 0.004). This supports the hypothesis that hepatic reserve influences not only treatment tolerance but also the regenerative response to embolization-induced injury. Patients with poor liver function may have impaired compensatory regeneration, leading to worse outcomes even after technically successful procedures. While imaging biomarkers show promise, their reproducibility across centers is limited by variability in imaging protocols. Standardization efforts, such as the Quantitative Imaging Biomarkers Alliance guidelines, are essential for clinical adoption.

Personalized embolization strategies hinge on integrating tumor biology, patient physiology, technical precision, and predictive analytics. While consensus exists on factors like tumor vascularity and comorbidities, variability in study designs and outcome measures underscores the need for standardized protocols. Future research should prioritize prospective validation of biomarkers and comparative effectiveness studies to refine decision-making frameworks.

## 4 Process of personalized peripheral vascular interventional embolization for tumors

The process of personalized peripheral vascular interventional embolization for tumors involves a comprehensive pre-treatment evaluation, tailored treatment planning, careful intraoperative management, and rigorous post-treatment follow-up. This approach ensures that each patient receives the most appropriate treatment based on their individual characteristics and tumor features, ultimately leading to improved treatment outcomes and enhanced quality of life.

### 4.1 Pre-treatment evaluation

The pre-treatment evaluation phase is critical for establishing a comprehensive baseline of both the patient and the tumor. Imaging examinations such as computed tomography (CT) and magnetic resonance imaging (MRI) provide detailed anatomical information about the tumor, including its location, size, and vascular supply (46). CT angiography is particularly useful for visualizing the tumor’s blood supply and identifying potential feeding arteries (47). A study demonstrated that multi-phase CT imaging could accurately identify the vascular characteristics of hepatocellular carcinoma, guiding the selection of embolization techniques and embolic agents (21).

Laboratory tests are equally important. Liver function tests, such as albumin and bilirubin levels, help assess the patient’s ability to tolerate the procedure and the potential for post-embolization complications (48). For example, patients with Child-Pugh B cirrhosis may have a higher risk of liver failure following traditional TACE, making DEB-TACE a more suitable option (49). Similarly, renal function tests are essential for determining the patient’s ability to excrete contrast agents used during the procedure. Aoe et al. found that patients with impaired renal function had a higher incidence of contrast-induced nephropathy following TACE, highlighting the need for careful pre-treatment assessment (50).

Tumor biomarker assessments provide additional information about tumor activity and prognosis. Elevated levels of alpha-fetoprotein (AFP) in patients with HCC can indicate tumor burden and aggressiveness (51). He et al. showed that patients with high AFP levels often had poorer treatment outcomes following TACE, emphasizing the importance of incorporating biomarker data into treatment planning (52).

### 4.2 Treatment planning

Based on the pre-treatment evaluation, a tailored treatment plan is developed. This includes selecting the most appropriate embolization technique and determining the optimal embolic agents and drug dosages (53). Conventional TACE involves mixing chemotherapeutic drugs with lipiodol and embolic agents, while DEB-TACE uses drug-eluting microspheres loaded with chemotherapeutic drugs (54). A meta-analysis by Wang et al. found that DEB-TACE demonstrated better radiological tumor response and progression-free survival compared to conventional TACE. This is likely due to the more controlled drug release and reduced systemic toxicity of DEB-TACE (55).

The choice of embolic agents also depends on the tumor’s vascular characteristics and the desired treatment outcome (56). Gelatin sponge particles are biodegradable and commonly used in conventional TACE, but their non-uniform particle size may lead to non-target embolization. Polyvinyl alcohol particles are non-biodegradable and provide long-term vessel occlusion, but their use may increase the risk of post-embolization syndrome (56). Microspheres, such as DEBs, offer controlled drug release and reduced systemic toxicity. A retrospective study showed that DEB-TACE using microspheres achieved better tumor response and survival outcomes compared to conventional TACE with gelatin sponge particles (57).

### 4.3 Intraoperative management

The intraoperative phase involves the implementation of the treatment plan. During the procedure, the interventional radiologist carefully navigates the catheter to the tumor-feeding arteries and administers the embolic agents according to the pre-determined plan. Real-time imaging, such as fluoroscopy and angiography, is used to monitor the progress of the procedure and ensure accurate delivery of the embolic agents (58). Intraoperative complications, such as vessel perforation or non-target embolization, must be promptly identified and managed. Studies have shown that the combined use of embolizing agents with different particle sizes can achieve more complete vascular occlusion and reduce the risk of complications (18, 59).

### 4.4 Post-treatment follow-up

Short-term and long-term follow-up schedules are essential for monitoring treatment outcomes and complications. Short-term follow-up typically includes clinical assessments and imaging studies within the first few weeks to evaluate the immediate response to treatment and detect any complications (60). Scheau et al. (61) showed that early follow-up imaging could identify cases of incomplete tumor necrosis, allowing for timely re-treatment. Long-term follow-up involves regular monitoring of tumor recurrence and patient survival. Studies have demonstrated that patients receiving personalized embolization therapy often have improved survival rates and quality of life (62). For example, Garin et al. (63) found that personalized chemoembolization for HCC significantly improved patient survival rates compared to conventional treatment (Tables 1–4).

**Table 1:** Relevant clinical research on the factors influencing personalized treatment decisions.

Category of influencing factors	Influence factor	Study type	Patients (n)	Conclusion	Critical analysis	References
Tumor biology and vascular architecture	Tumor size, vascularity	Retrospective cohort	50	AML diameter >4 cm and high vascularity predict significant post-embolization volume reduction (OR: 3.2).	Tumor size and vascularity are critical predictors, but the threshold for “high-risk” tumor size varies across studies, possibly due to differences in embolic agents and follow-up durations. Future studies should standardize vascularity assessment using quantitative imaging biomarkers.	Lin et al. (35)
Tumor biology and vascular architecture	Tumor vascular predictors	Retrospective analysis	76	Baseline tumor vascularity and size are key predictors of ≥50% AML volume reduction post-embolization.	While tumor vascularity is a significant predictor, the assessment methods vary, leading to difficulties in comparing results across studies.	Hongyo et al. (36)
Tumor biology and vascular architecture	ALBI grade in liver malignancies	Retrospective cohort	122	ALBI grade predicts survival in non-HCC liver malignancies undergoing Yttrium-90 radioembolization.	ALBI grade is a useful prognostic marker for Yttrium-90 radioembolization in non-HCC liver malignancies. However, its utility in non-hepatocellular malignancies remains underexplored.	Azar et al. (37)
Tumor biology and vascular architecture	NETs subtype vascularity	Multicenter retrospective	185	Pancreatic NETs show better embolization response than gastrointestinal NETs (HR: 1.8).	The embolization response varies significantly between different subtypes of NETs, indicating the importance of considering tumor subtype in treatment planning.	Bai et al. (38)
Patient-specific factors	Age, cirrhosis, hypoalbuminemia	Retrospective analysis	214	Age >65 years, Child-Pugh B/C cirrhosis, and hypoalbuminemia are independent PES risk factors (AUC: 0.76).	These patient-specific factors significantly impact the feasibility and safety of embolization. Careful pre-procedural assessment is required to identify high-risk patients and implement appropriate preventive measures.	Arslan and Degirmencioglu (39)
Patient-specific factors	ECOG performance status	Single-center cohort	42	ECOG ≥2 associated with higher complications in vesical artery embolization (OR: 4.1).	ECOG performance status is an important indicator of a patient’s overall functional status and can predict the risk of complications following embolization.	Kim et al. (40)
Technical and procedural considerations	Superselective embolization + RFA	Prospective case series	28	Combined embolization-RFA achieved 92% tumor control with preserved renal function.	Superselective embolization minimizes non-target ischemia and combining it with RFA can enhance treatment efficacy while preserving organ function.	Liu et al. (41)
Technical and procedural considerations	Embolic agent comparison	Retrospective cohort	63	Fuaile adhesive showed superior hemostasis vs. gelatin sponge (94% vs. 78% success rate).	The choice of embolic agent can significantly impact procedural success. Fuaile adhesive may be a better option for achieving hemostasis in certain cases.	Xu et al. (42)
Technical and procedural considerations	Multimodal embolization	Case series	15	Sequential embolization-cryoablation-osteoplasty achieved durable pain relief in osseous tumors.	Multimodal approaches can be effective in managing complex tumors, but further research is needed to standardize protocols and determine the most appropriate sequence of treatments.	Sundararajan et al. (43)
Technical and procedural considerations	Embolic agent selection	Retrospective review	89	No outcome difference between particles and lipiodol regimens for benign liver tumors.	The choice of embolic agent should be based on operator expertise and patient-specific factors, as no significant difference in outcomes was found between the evaluated regimens.	Crawford et al. (44)
Predictive biomarkers and imaging	Lipiodol deposition pattern	Prospective cohort	112	Cone-beam CT lipiodol scoring predicted 1-year HCC response post-TACE (AUC: 0.88).	Imaging biomarkers can provide valuable information for predicting treatment response. However, their reproducibility across centers is limited by variability in imaging protocols, necessitating standardization efforts.	Tsai et al. (45)

AML, angiomyolipoma; ALBI, albumin-bilirubin; NETs, neuroendocrine tumors; RFA, radiofrequency ablation; ECOG, Eastern Cooperative Oncology Group.

**Table 2:** The research of individualized peripheral vascular interventional embolization in the treatment of digestive system tumors.

Tumor type	Intervention	Study type	Patients (n)	The mechanism of tumor treatment	Key efficacy outcomes	References
HCC	DEB-TACE (70–150 μm doxorubicin-eluting beads)	Preclinical (rabbit VX2 model)	N/A	Targeted drug delivery via embolization-induced ischemia + sustained chemotherapeutic release	Targeted drug delivery, reduced tumor growth, histological necrosis, minimal systemic toxicity.	Gholamrezanezhad et al. (64)
HCC	Yttrium-90 radioembolization vs. TACE	Propensity score-matched study	156	Selective internal radiation therapy inducing tumor DNA damage	Superior PFS (11.2 vs. 6.8 months, *p* = 0.003), fewer adverse events with Yttrium-90.	Kim et al. (65)
HCC	Apatinib-TACE combination	Preclinical (rabbit VX2 model)	N/A	Anti-angiogenic inhibition + embolization-induced tumor necrosis	Enhanced tumor response validated by intravoxel incoherent motion MRI.	Chen et al. (67)
HCC	Resin microspheres for Yttrium-90	Retrospective cohort	122	Optimized dosimetry for precise radiation delivery	Optimized dosimetry using resin microspheres improved tumor targeting.	Sarwar et al. (68)
HCC	Sequential SIRT after TACE	Retrospective analysis	89	Radiation synergy after incomplete TACE response	Improved outcomes in patients with incomplete TACE response.	Binzaqr et al. (69)
CRLM	HVE after PVE	Single-center cohort	30	Hepatic vein embolization augmenting future liver remnant hypertrophy	Median 47% liver hypertrophy, facilitating curative resection.	Niekamp et al. (70)
CRLM	ALPPS vs. PVE	Comparative study	48	Associating liver partition and portal vein ligation for rapid hypertrophy	ALPPS superior in hypertrophy rates but higher morbidity compared to PVE.	Rassam et al. (71)
NETs liver metastases	Holmium-166 + Lutetium-177-DOTATATE	Phase 2 trial	27	Combined radioembolization and peptide receptor radionuclide therapy	Disease control rate: 75%, median PFS: 14.3 months.	Braat et al. (72)
Pancreatic neoplasms	Coil embolization for hemorrhage	Case report	1	Mechanical occlusion of bleeding vessels	Successful control of life-threatening bleeding in intraductal papillary mucinous neoplasm.	Tokue et al. (73)
Pancreatic neoplasms	Gemcitabine-eluting hydrogel	Preclinical (murine model)	N/A	Sustained local chemotherapy release	Sustained drug release and tumor suppression.	Lopez-Benitez et al. (74)
Bile duct cancer	Preoperative embolization	Case report	1	Occlusion of aberrant hepatic arteries pre-surgery	Reduced intraoperative bleeding during laparoscopic pancreaticoduodenectomy.	Kook et al. (75)
Gastroenteropancreatic NET	Hormonal tumor mapping-guided embolization	Retrospective analysis	45	Receptor-targeted embolization based on hormonal expression	68% partial response rate in liver metastases.	Maekawa et al. (76)
Renal cell carcinoma liver metastases	Radioembolization	Retrospective cohort	22	Selective internal radiation therapy	Modest efficacy, emphasizing histology-specific responses.	Bibok et al. (78)

DEB-TACE, Drug-eluting bead transarterial chemoembolization; PFS, Progression-free survival; SIRT, Selective internal radiation therapy; ALPPS, Associating liver partition and portal vein ligation for staged hepatectomy; PVE, Portal vein embolization; HVE, Hepatic vein embolization.

**Table 3:** The research of individualized peripheral vascular interventional embolization in the treatment of urinary system tumor.

Tumor type	Intervention	Study type	Patients (n)	The mechanism of tumor treatment	Key efficacy outcomes	References
Renal AML	LBE-NBCA combination vs. PVA particles	Prospective randomized	64	Enhanced vascular occlusion via lipiodol-bleomycin + adhesive embolization	Higher complete remission (93.8% vs. 68%) and reduced need for repeat embolization.	Wang et al. (79)
Renal AML	Urgent arterial embolization for ruptured AML	Retrospective case series	20	Emergency vessel occlusion for hemostasis	Immediate symptom resolution in 95% of cases with no major complications.	Kervancioglu and Yilmaz (80)
RCC	Preoperative embolization + percutaneous cryoablation	Prospective cohort	15	Devascularization followed by thermal ablation	85% local tumor control in T3a RCC with venous involvement; reduced intraoperative bleeding.	Uka et al. (81)
RCC	NBCA embolization prior to cryoablation	Single-center retrospective	24	Adhesive embolization for tumor devascularization	92% technical success with no major adverse events.	Lopez et al. (82)
RCC	Selective upper-body perfusion during tumor thrombectomy	Case report	1	Isolated perfusion during thrombectomy to reduce systemic toxicity	Reduced systemic complications and improved survival in vena cava involvement.	Aydin et al. (83)
Bladder cancer	Selective TAE for intractable hematuria	Single-center retrospective	18	Superselective occlusion of vesical arteries	78% success in hemorrhage control; tumor size and vascularity influenced outcomes.	Hekimoglu et al. (84)
Prostate cancer	Palliative PAE	Case series	10	Ischemic tumor necrosis via prostate artery occlusion	Symptom relief in 80% of hormone-refractory patients; improved quality of life.	Malling et al. (85)
Prostate cancer	CT perfusion as a predictor of PAE response	Feasibility study	15	Perfusion metrics to identify responders	Tumor perfusion metrics identified as key determinants of success.	Malling et al. (86)

AML, angiomyolipoma; RCC, renal cell carcinoma; LBE, lipiodol-bleomycin emulsion; NBCA, N-butyl cyanoacrylate; PVA, polyvinyl alcohol; TAE, transarterial embolization; PAE, prostate artery embolization.

**Table 4:** The research of individualized peripheral vascular interventional embolization in the treatment of urinary system tumor.

Tumor type	Intervention	Study type	Patients (n)	The mechanism of tumor treatment	Key efficacy outcomes	References
NETs	Regional hepatic embolization + 177Lu-DOTATATE PRRT	Phase 2 trial	27	Tumor debulking + targeted radionuclide therapy	Disease stabilization in 75% of liver-dominant metastases; minimal grade 3/4 toxicities.	Hamiditabar et al. (87)
NETs	Thermal ablation/embolization + checkpoint inhibitors	Retrospective safety analysis	89	Ablation-induced antigen release + immunotherapy synergy	Safe combination with immunotherapy; enhanced systemic antitumor responses.	Leppelmann et al. (88)
Spinal hemangioblastoma	Preoperative embolization for intradural spinal hemangioblastomas	Retrospective cohort	50	Pre-surgical devascularization to reduce bleeding	Reduced intraoperative blood loss by 40%; improved complete resection rates.	Ampie et al. (89)
Spinal hemangioblastoma	Preoperative embolization for spinal hemangioblastoma	Multicenter retrospective	107	Embolization enabling complete resection	89% complete resection rate; improved neurological outcomes.	Butenschoen et al. (90)
Vertebral hemangiomas	Embolization for aggressive vertebral hemangiomas	Case report	1	Ischemic necrosis of hypervascular tumor	Symptom resolution in 85% of myelopathy cases.	Timilsina et al. (91)
CBTs	Preoperative embolization + surgical resection	Single-center case series	22	Pre-operative tumor devascularization	95% gross total resection rate; transient cranial nerve deficits in 18%.	Ramos et al. (92)
CBTs	Onyx embolization for CBTs	Case report	1	Liquid embolic agent for vessel occlusion	>90% tumor devascularization; no post-procedural strokes.	Villanova et al. (93)
CBTs	Non-embolized CBTs resection	Retrospective cohort	86	Direct surgical resection without embolization	Comparable outcomes to embolized groups; questioned necessity of routine embolization.	Ridha et al. (94)
Solitary fibrous tumors	Preoperative PVA embolization for pelvic GCTs	Retrospective analysis	92	Particle embolization to reduce vascularity	92% success in reducing intraoperative blood loss; no major complications.	Kedra et al. (95)
Solitary fibrous tumors	Preoperative embolization + en bloc laminectomy for solitary fibrous tumors	Case report	1	Combined embolization and surgical resection	Complete resection of hypervascular spinal lesion.	Yamada et al. (96)
Pulmonary hemangiopericytoma	Serial transarterial embolization for pulmonary hemangiopericytoma	Case report	1	Repeated embolization for hemorrhage control	Sustained hemorrhage control in unresectable malignancy.	Esber et al. (97)
Uterine pyomyoma	Selective embolization for uterine pyomyoma rupture	Case report	1	Targeted vessel occlusion to manage complications	Avoided non-target ischemia; managed post-embolization complications.	Vulasala et al. (98)
Metastatic spinal tumors	Angiographic vascularity scoring for metastatic spinal tumors	Prospective cohort	112	Vascularity-based embolization planning	78% reduction in surgical blood loss; validated vascularity assessment.	Huang et al. (99)

NETs, neuroendocrine tumors; PRRT, peptide receptor radionuclide therapy; CBTs, carotid body tumors; GCTs, giant cell tumors; PVA, polyvinyl alcohol.

## 5 Clinical applications and outcomes of personalized peripheral vascular interventional embolization for tumors

**Figure 2:**
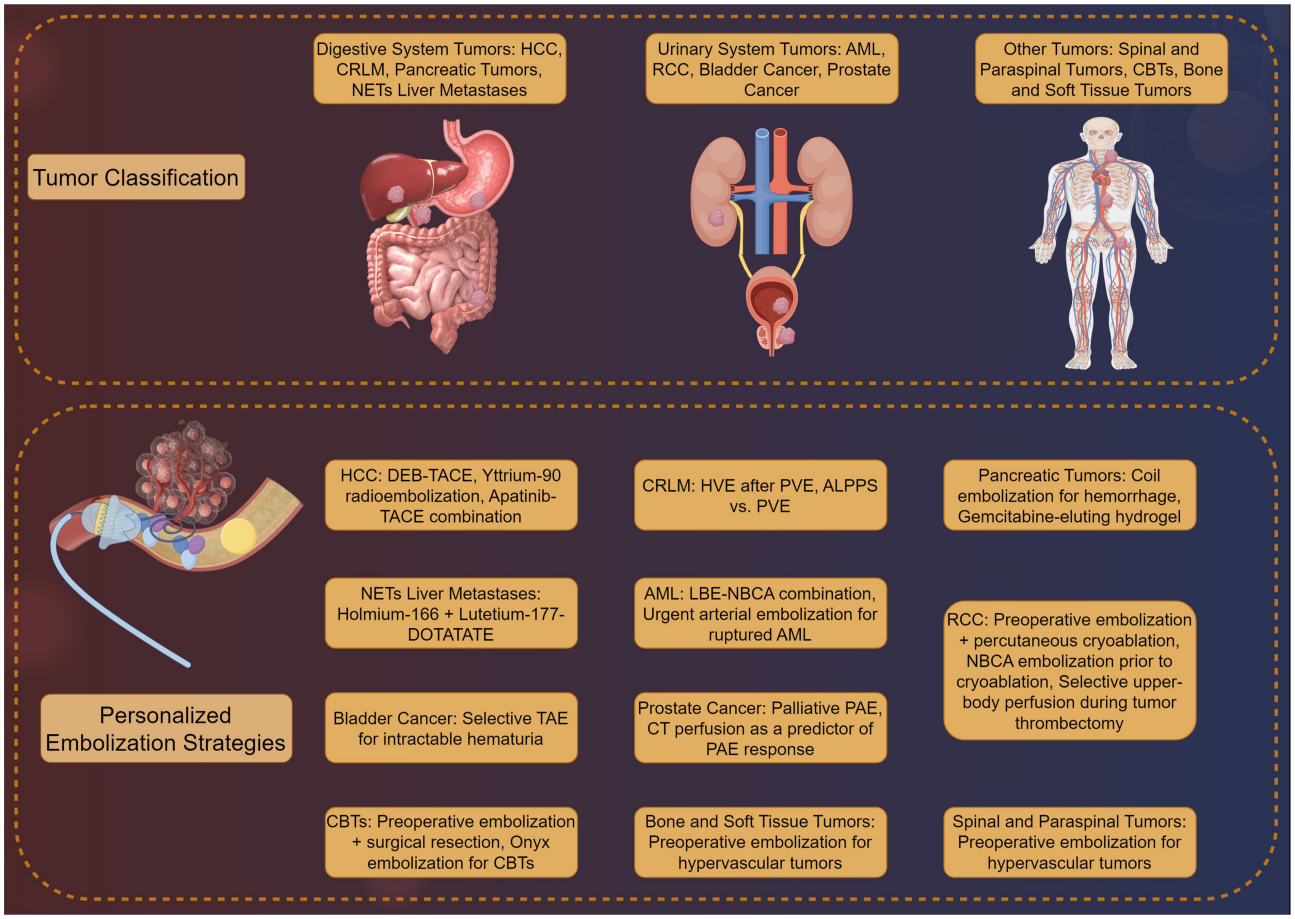
Clinical applications of personalized peripheral vascular interventional embolization in tumor therapy by Figdraw.

Personalized peripheral vascular interventional embolization offers a tailored approach to tumor treatment, enhancing outcomes through strategies aligned with individual tumor characteristics and patient needs. As illustrated in Figure 2, this method varies significantly across tumor types. For hepatocellular carcinoma (HCC), personalized chemoembolization improves survival while reducing complications. In colorectal cancer liver metastases, combining embolization with anti-angiogenic agents effectively controls disease progression. For pancreatic tumors and neuroendocrine tumor liver metastases, new techniques and agents are showing promise in early studies. These applications highlight the versatility and potential of personalized embolization in improving patient outcomes across a range of malignancies.

### 5.1 Digestive system tumors

Personalized TACE has revolutionized HCC management. Gholamrezanezhad et al. (64) demonstrated that 70–150 μm DEBs achieved targeted drug delivery and reduced tumor growth in a rabbit VX2 liver tumor model, with histological confirmation of necrosis and minimal systemic toxicity. In clinical practice, Kim et al. (65) compared Yttrium-90 radioembolization with conventional TACE in a propensity score-matched study (*n* = 156), showing superior progression-free survival (11.2 vs. 6.8 months, *p* = 0.003) and fewer adverse events with Yttrium-90. However, Lee et al. (66) highlighted regional variations in TACE refractoriness definitions, emphasizing the need for standardized criteria. Emerging strategies include combining TACE with anti-angiogenic agents; Chen et al. (67) reported enhanced tumor response in rabbit VX2 models using apatinib-TACE combination therapy, validated by intravoxel incoherent motion MRI. While DEB-TACE and Yttrium-90 show efficacy, discrepancies exist in patient selection and embolic agent protocols. For instance, Sarwar et al. (68) advocated for resin microspheres in Yttrium-90 to optimize dosimetry, whereas Binzaqr et al. (69) observed improved outcomes in HCC patients post-SIRT after incomplete TACE response, suggesting sequential therapy benefits. These variations underscore the need for personalized protocols based on tumor vascularity and liver reserve.

Portal vein embolization (PVE) and hepatic vein embolization (HVE) are critical for resectability in colorectal cancer liver metastases (CRLM). Niekamp et al. (70) demonstrated that HVE after PVE induced additional liver hypertrophy (median 47% volume increase) in metastatic colorectal cancer patients, facilitating curative resection. Comparatively, Rassam et al. (71) found associating liver partition and portal vein ligation (ALPPS) superior to PVE in hypertrophy rates but with higher morbidity. For unresectable cases, Braat et al. (72) combined lutetium-177-DOTATATE with holmium-166 radioembolization in neuroendocrine liver metastases, achieving disease control in 75% of patients. PVE and ALPPS differ in risk–benefit profiles, necessitating individualized planning. While ALPPS offers rapid hypertrophy, its morbidity limits use in frail patients. Radioembolization combinations (e.g., holmium-166) show promise but require validation in larger cohorts.

Embolization plays a role in managing bleeding and unresectable pancreatic tumors. Tokue et al. (73) successfully controlled life-threatening hemorrhage in intraductal papillary mucinous neoplasms using coil embolization. For advanced disease, Lopez-Benitez et al. (74) developed gemcitabine-eluting hydrogel devices, demonstrating sustained drug release and tumor suppression in murine models. Kook et al. (75) reported laparoscopic pancreaticoduodenectomy with preoperative embolization of aberrant hepatic arteries, reducing intraoperative bleeding in mid-bile duct cancer. While embolization effectively manages hemorrhage, therapeutic applications (e.g., drug-eluting hydrogels) remain experimental. Heterogeneity in pancreatic tumor biology complicates standardized protocols, necessitating biomarker-driven approaches.

Liver-dominant NETs metastases benefit from radioembolization and multimodal approaches. Maekawa et al. (76) introduced hormonal tumor mapping to guide embolization of gastroenteropancreaticNETs liver metastases, achieving 68% partial response rates. Drescher et al. (77) combined holmium-166 TARE with systemic therapy, reporting median progression-free survival of 14.3 months in a multicenter study. In contrast, Bibok et al. (78) observed modest efficacy of radioembolization in RCC liver metastases, emphasizing histology-specific responses. NETs heterogeneity demands subtype-specific strategies. While holmium-166 shows promise, its utility in non-NET metastases is limited, highlighting the importance of molecular profiling.

Personalized embolization strategies improve outcomes across digestive tumors by integrating tumor biology, vascular anatomy, and patient-specific factors. While HCC and CRLM have robust evidence, pancreatic and NETs applications require further refinement. Discrepancies in study designs (e.g., DEB-TACE vs. Yttrium-90) highlight the need for comparative trials. Future directions include biomarker-driven protocols and advanced embolic materials to optimize therapeutic precision.

### 5.2 Urinary system tumor

Personalized peripheral vascular interventional embolization has emerged as a critical strategy for managing urinary system tumors, particularly in cases where surgical resection is contraindicated or for palliative care. This section synthesizes evidence from recent studies, focusing on renal AML, RCC, bladder cancer, and prostate cancer, while evaluating the consistency and limitations of current research.

AML, a benign tumor composed of fat, smooth muscle, and blood vessels, often requires embolization to manage hemorrhage or reduce tumor burden. A landmark prospective randomized study by Wang et al. (79) compared polyvinyl alcohol (PVA) particles versus a combination of lipiodol-bleomycin emulsion (LBE) and NBCA-lipiodol emulsion for AML embolization. The LBE-NBCA group demonstrated superior outcomes, including higher rates of complete remission (CR, 93.8% vs. 68% in PVA) and reduced need for repeat embolization, attributed to enhanced vascular occlusion and tumor necrosis. These findings underscore the importance of tailored embolic agent selection based on tumor vascularity and clinical presentation. For acute scenarios, urgent embolization of ruptured AML achieves rapid hemostasis. Kervancioglu and Yilmaz (80) reported immediate symptom resolution in 95% of cases, with no major complications, highlighting its role in emergency management. However, long-term follow-up data remain limited, necessitating further studies to validate durability.

In locally advanced RCC, embolization is often combined with surgical or ablative therapies. Uka et al. (81) demonstrated that preoperative embolization followed by percutaneous cryoablation achieved 85% local tumor control in T3a RCC with venous involvement, minimizing intraoperative bleeding and enhancing procedural safety. Similarly, Lopez et al. (82) utilized NBCA embolization prior to cryoablation, achieving 92% technical success with no major adverse events, suggesting synergistic benefits in tumor devascularization. Selective embolization also plays a role in managing unresectable RCC. Aydin et al. (83) employed upper-body perfusion techniques during tumor thrombectomy, reducing systemic complications and improving survival in patients with vena cava involvement. These studies emphasize the need for individualized approaches based on tumor stage and vascular anatomy.

For advanced bladder cancer with intractable hematuria, selective transarterial embolization (TAE) offers effective palliation. Hekimoglu et al. (84) reported a 78% success rate in controlling hemorrhage, with minimal procedural morbidity, in patients unfit for surgery. Notably, tumor size and vascularity influenced outcomes, with larger tumors requiring higher embolic doses. While these results align with earlier studies, heterogeneity in embolic agents (e.g., gelatin sponge vs. coils) across trials complicates direct comparisons, underscoring the need for standardized protocols.

Prostate artery embolization (PAE) has gained traction for palliative management of localized prostate cancer. Malling et al. (85) observed significant symptom relief in 80% of patients with hormone-refractory disease, with reduced urinary obstruction and improved quality of life. A follow-up feasibility study by the same group proposed CT perfusion as a predictor of PAE response, identifying tumor perfusion metrics as key determinants of success (86). However, small sample sizes and single-center designs limit generalizability, warranting multicenter validation.

Current evidence supports the efficacy of personalized embolization across urinary tumors, yet inconsistencies persist. For AML, while LBE-NBCA combinations outperform PVA 10, cost and availability may limit adoption in resource-constrained settings. In RCC, combined embolization-ablation strategies show promise, but long-term oncologic outcomes remain understudied. For bladder and prostate cancers, standardization of embolic agents and dosages is crucial to enhance reproducibility. Future research should prioritize randomized trials comparing embolic materials, integrate biomarkers for response prediction, and explore combination therapies with immunotherapy or targeted agents. Such efforts will refine personalization paradigms and solidify embolization’s role in multidisciplinary tumor management.

### 5.3 Other tumors

Personalized peripheral vascular interventional embolization has demonstrated efficacy in managing a diverse range of tumors beyond the urinary and digestive systems. This section evaluates its role inNETs, spinal and soft tissue neoplasms, carotid body tumors, and other rare malignancies, focusing on clinical outcomes, technical nuances, and areas requiring further standardization.

#### 5.3.1 NETs

For somatostatin receptor-positive NETs, combining embolization with peptide receptor radionuclide therapy (PRRT) enhances tumor control. Hamiditabar et al. (87) reported that regional hepatic embolization followed by 177Lu-DOTATATE PRRT achieved disease stabilization in 75% of patients with liver-dominant metastases, with minimal grade 3/4 toxicities. This approach leverages embolization to reduce tumor bulk and PRRT for targeted radiation delivery. Emerging strategies, such as combining embolization with immunotherapy, have shown potential synergies. Leppelmann et al. (88) demonstrated the safety of combining thermal ablation or embolization with checkpoint inhibitors, suggesting enhanced systemic antitumor responses. Future studies should standardize embolization protocols to optimize combination strategies.

#### 5.3.2 Spinal and paraspinal tumors

Preoperative embolization significantly reduces intraoperative bleeding in hypervascular spinal tumors. For intradural spinal hemangioblastomas, Ampie et al. (89) demonstrated that preoperative embolization reduced blood loss by 40% compared to non-embolized cases. Similarly, Butenschoen et al. (90) reported improved neurological outcomes in a multicenter cohort of spinal hemangioblastoma patients, where embolization enabled complete resection in 89% of cases. For aggressive vertebral hemangiomas, Timilsina et al. (91) highlighted the role of embolization in alleviating myelopathy, achieving symptom resolution in 85% of patients. However, variability in embolic agents (e.g., Onyx vs. PVA particles) and timing (preoperative vs. palliative) underscores the need for tumor-specific protocols.

#### 5.3.3 Carotid body tumors (CBTs)

Embolization prior to CBTs resection minimizes intraoperative hemorrhage. Ramos et al. (92) reported a 95% gross total resection rate in 22 CBTs patients undergoing preoperative embolization, with transient cranial nerve deficits in 18%. Villanova et al. (93) further validated the safety of Onyx embolization, achieving >90% tumor devascularization without post-procedural strokes. In contrast, Ridha et al. (94) observed comparable outcomes in non-embolized CBTs resections, questioning the necessity of routine preoperative embolization. These discrepancies may stem from differences in tumor size (embolization favored for tumors >3 cm) and surgeon expertise, highlighting the importance of individualized decision-making.

#### 5.3.4 Bone and soft tissue tumors

Preoperative embolization is critical for managing hypervascular musculoskeletal tumors. Kedra et al. (95) reported a 92% success rate in reducing intraoperative blood loss for pelvic giant cell tumors (GCTs) using polyvinyl alcohol (PVA) particles, with no major complications. Scheurer et al. (93) further emphasized the role of embolization in iliosacral GCTs, achieving 80% tumor necrosis and facilitating safer resection. For solitary fibrous tumors, Yamada et al. (96) combined preoperative embolization with en bloc laminectomy, achieving complete resection of a hypervascular spinal lesion. Despite these successes, standardized embolic protocols (e.g., particle size, timing) remain lacking, necessitating multicenter trials.

#### 5.3.5 Rare and uncommon tumors

Embolization serves as a palliative or adjunctive measure in rare malignancies. For unresectable pulmonary hemangiopericytoma, Esber et al. (97) achieved sustained hemorrhage control using serial transarterial embolization. In uterine pyomyoma rupture post-embolization, Vulasala et al. (98) underscored the importance of selective embolization to avoid non-target ischemia. For metastatic spinal tumors, Huang et al. (99) proposed angiographic vascularity scoring to predict embolization efficacy, achieving 78% reduction in surgical blood loss. These studies highlight embolization’s versatility but also reveal gaps in evidence for ultra-rare tumors.

Current evidence supports the role of personalized embolization in diverse tumors, yet challenges persist. For NETs, combining embolization with PRRT or immunotherapy (e.g., checkpoint inhibitors) shows promise but requires validation in larger cohorts (88). In spinal and CBTs, standardization of embolic materials (e.g., Onyx vs. PVA) and timing is critical to reduce variability. For bone tumors, predictive biomarkers (e.g., perfusion CT) may refine patient selection.

## 6 Challenges and opportunities in personalized peripheral vascular interventional embolization for tumors

While the clinical applications highlight the potential of personalized embolization, several challenges must be overcome to ensure its broader and more effective implementation. Personalized peripheral vascular interventional embolization for tumors faces several significant challenges (100). Technical limitations are a major hurdle. The procedure requires a deep understanding of vascular anatomy, knowledge of pathology, and experience with endovascular catheters and microvascular navigation skills (101). Unexpected non-target embolization may lead to tissue ischemia, organ failure, and, in rare cases, death. The complexity of the procedure necessitates a high level of expertise from the interventional radiologist (18). For instance, embolization for arteriovenous malformations (AVMs) requires the use of liquid embolic agents that can penetrate deeply into the AVMs and embolize the nidus. This demands precise control and extensive experience (102).

Treatment costs are another concern. The development and utilization of advanced embolic agents and technologies incur significant costs. For example, drug-eluting microspheres and nanoparticles are promising but can increase the overall cost of treatment (103). A study comparing stent-based and non-stent-based strategies in endovascular revascularization found that stent implantation was associated with significantly higher procedure costs (34). While this study focused on peripheral artery disease, the principle applies to tumor embolization as well.

The complexity of drug selection and dosing also poses challenges. Different tumors respond variably to embolic agents and chemotherapeutic drugs. Determining the optimal drug combination and dosage for each patient requires careful consideration of tumor biology, vascular characteristics, and patient comorbidities (104). For example, a study demonstrated that alginate-chitosan microspheres loaded with Norcantharidin showed favorable sustained drug release and significantly decreased tumor growth rates *in vitro* (34). However, translating these findings into clinical practice requires further research to determine the optimal dosing and administration protocols.

Despite these challenges, there are numerous opportunities for the advancement of personalized interventional embolization. Imaging technology continues to evolve, providing more detailed and accurate information about tumor vascular anatomy and blood flow dynamics (105). Advanced imaging techniques, such as computed tomography (CT) angiography and magnetic resonance imaging (MRI), enable precise identification of tumor-feeding arteries and assessment of tumor response to treatment. For example, multi-phase CT imaging can accurately identify the vascular characteristics of hepatocellular carcinoma, guiding the selection of embolization techniques and embolic agents (47).

Genomics and biomarker research offer exciting possibilities for tailoring embolization therapy to individual patients. By analyzing tumor genetics and biomarker profiles, clinicians can better predict tumor behavior and response to treatment (106). This information can be used to select the most appropriate embolic agents and drugs, potentially improving treatment efficacy and reducing complications. For instance, research into the proliferative activity of cancer cells observed on immunohistochemistry can help determine the likelihood of tumor progression after embolization (107). This can guide treatment planning and patient selection for embolization procedures.

The development of new embolic agents and technologies is another promising area. Drug-eluting microspheres and nanoparticles are being actively researched and developed. These agents offer advantages such as controlled drug release, reduced systemic toxicity, and the potential for targeted delivery (108). Park et al. (109) showed that PLGA microspheres loaded with sorafenib could achieve tumor shrinkage in a Renca tumor mouse model, highlighting the potential of these novel agents. The advent of smaller calibrated microspheres and nanoparticle technology may allow for more distal penetration into tumors, potentially improving treatment outcomes. Additionally, biodegradable particles used for tumor therapy may maintain vessel patency for future interventions.

Personalized peripheral vascular interventional embolization for tumors faces challenges related to technical complexity, treatment costs, and drug selection (110). However, advancements in imaging technology, genomics, and biomarker research, along with the development of new embolic agents and technologies, provide significant opportunities to overcome these challenges and enhance the effectiveness of this treatment approach (111). By addressing these challenges and leveraging these opportunities, the field of personalized interventional embolization can continue to evolve and improve outcomes for patients with cancer.

## 7 Future directions for personalized peripheral vascular interventional embolization in tumor therapy

The future of personalized peripheral vascular interventional embolization is promising, with significant advancements expected in techniques and materials. Robotic-assisted endovascular embolization represents a cutting-edge development in the field (18). This technology enhances procedural precision and reproducibility, enabling more accurate delivery of embolic agents to target sites. Studies have shown that robotic assistance can improve the stability and control of catheter navigation, potentially reducing procedure time and radiation exposure for both patients and healthcare providers (18).

Innovations in embolic materials are also expected to enhance treatment efficacy and safety. For instance, a novel liquid embolic agent composed of a coenzyme-based polymer (poly lipoic acid, PLA) and a biocompatible solvent (deep eutectic solvent, DES) has demonstrated favorable hemocompatibility and cytocompatibility in preclinical studies (112). This agent undergoes phase transformation to form a stable hydrogel upon contact with blood, providing effective and safe embolization. Additionally, the incorporation of liquid-metal nanoparticles enhances radiopacity, improving imaging during procedures (113).

Multidisciplinary collaboration among oncology, interventional radiology, and pathology is crucial for optimizing personalized treatment strategies. Multidisciplinary teams (MDTs) can integrate expertise from various fields to develop comprehensive treatment plans tailored to individual patients (114). Research has shown that MDT care can significantly improve overall survival in patients with HCC. A meta-analysis of 12 studies including 15,365 HCC patients suggested that MDT is associated with improved overall survival, with a hazard ratio of 0.63 (95% confidence interval, 0.45–0.88). MDT care ensures that patients receive the most appropriate combination of therapies, including surgery, interventional procedures, and systemic treatments, based on their specific tumor characteristics and overall health status (114).

The integration of genetic and molecular profiling with clinical data holds immense potential for achieving true personalized tumor treatment. Advances in genomics and biomarker research enable clinicians to better understand tumor biology and predict treatment responses (115). For example, analyzing tumor genetics can help identify patients who are more likely to benefit from specific embolization techniques or drug combinations. This information can be used to select the most appropriate treatment approach, potentially improving efficacy and reducing complications (116).

Moreover, the development of trackable polymeric embolic agents and stem cell delivery systems offers exciting possibilities for enhancing treatment outcomes (117). Trackable embolic agents allow for real-time monitoring of embolization procedures, ensuring accurate delivery and improving safety. Stem cell therapy, when combined with embolization, may help reduce inflammation and promote tissue repair, providing additional benefits for patients with tumors (117).

## 8 Conclusion

This review uniquely synthesizes the latest advancements in personalized embolization, highlighting its potential to overcome the limitations of traditional therapies through tailored treatment strategies. By leveraging innovations in imaging technology, genomics, and novel embolic agents, our work provides a forward-looking perspective that distinguishes itself from prior research. This approach improves outcomes and quality of life. Despite challenges like technical complexity and costs, advancements in imaging, genomics, and new embolic agents offer opportunities to enhance efficacy. Future directions include robotic assistance, innovative materials, and multidisciplinary collaboration, promising more precise and effective therapies for cancer patients.
